# Obese people benefit from lumbar spinal stenosis surgery as much as people of normal weight

**DOI:** 10.1186/s13018-021-02692-z

**Published:** 2021-09-07

**Authors:** Henni Myllykangas, Leena Ristolainen, Heikki Hurri, Jouni Lohikoski, Hannu Kautiainen, Ville Puisto, Heikki Österman, Mikko Manninen

**Affiliations:** 1 Orton Orthopaedic Hospital, Tenholantie 10, 00280 Helsinki, Finland; 2 Research Institute Orton, Helsinki, Finland; 3 Medcare, Espoo, Finland

**Keywords:** Spinal stenosis, Operative treatment, Obesity, Quality of life

## Abstract

**Background:**

Lumbar spinal stenosis (LSS) is a common degenerative condition of the spine that causes back pain radiating to the lower extremity. Surgical treatment is indicated to treat progressive radical symptoms. Obesity has been associated with inferior results in the domains of quality of life (QoL) following an LSS operation, but the research findings have been limited. This paper aims to identify whether obesity affects QoL due to back pain among patients who underwent an operation for LSS.

**Methods:**

This study is based on a series of patients operated on for LSS between 2012 and 2018. Operated patients who returned for follow-up forms within the first or second years were included. A total of 359 patients were selected, 163 males (45%) and 196 females (55%). The mean age was 68.9 years. The EuroQol five-dimension scale (EQ-5D) questionnaire was chosen to measure QoL and the Oswestry Disability Index (ODI) for functional disability.

**Results:**

QoL, as measured by EQ-5D, was preoperatively lower in those patients with a BMI ≥ 30. One year after the operation, all groups had a similar trend of improved QoL. At the second year, the results in all groups levelled off even though there was no statistical difference in clinical outcomes (*p* = 0.92).

The ODI was preoperatively statistically higher in patients with a BMI ≥ 30 (*p* < 0.001). Two years after the surgery, all groups had improved ODI scores, but there was no statistical difference in ODI between the BMI groups (*p* = 0.54).

**Conclusion:**

Surgical intervention for debilitating or longstanding symptoms of LSS should be considered as a treatment option for suitable patients in spite of an elevated BMI.

## Introduction

Lumbar spinal stenosis (LSS) is a degenerative process of the spine that leads to the progressive compression of the neural structures in the spinal canal. The causes can be congenital or acquired (degenerative), with the latter being more prevalent among the elderly. LSS can be classified based on the localization of compression in the spinal cord: (a) central stenosis, (b) lateral stenosis and (c) intervertebral foraminal stenosis.

Typical symptoms related to spinal stenosis are lower back pain that radiates to the leg (buttocks, hamstrings and calves), together with sensory and motor nerve symptoms (numbness and weakness of the legs). Symptoms are exacerbated by walking (referred to as neurogenic claudication), while they are eased by a forward bend and sitting, taking off pressure from the posterior spinal column [1]. The L4–L5 segment is most frequently affected by LSS [2].

Symptoms associated with LSS are generally treated conservatively, but operative treatment should be considered in cases of debilitating back and/or foot pain if radicular symptoms progress or with the acute onset of cauda equine syndrome. Surgery is also indicated if symptoms have not eased in 3–6 months despite adequate conservative treatment.

The goal of surgical treatment of LSS is the decompression of the nerves. Surgery is done in either an open, microscopic or endoscopic way. Selected patients may benefit from an additional arthrodesis. Earlier, the addition of arthrodesis to neural decompression surgery was suggested in the case of coexisting spondylolisthesis. However, research findings are controversial, and there are no clear guidelines on when arthrodesis should be performed [3].

Numerous researchers have aimed to identify patient-related preoperative predictors for decompressive laminectomy in LSS surgery. Multiple preoperative factors, most importantly leg pain exceeding 2 years and preoperative functional ability, influence outcomes in spinal stenosis surgery [4]. In a systematic review of 21 internationally published articles by Aalto et al. [5], factors predicting positive outcomes were better walking ability, preoperative mental and somatic well-being, and higher income level. Negative predictive factors were smoking, depression and high cardiovascular comorbidity.

Obesity has been associated with a worse prognosis following LSS surgery and an increased incidence of postoperative complications, such as infections, but the research findings have been controversial [6–8]. This paper aims to identify whether obesity affects the QoL and functional disability due to back pain over a 2-year follow-up period for patients who underwent an operation for LSS at Orton Orthopaedic Hospital in Finland.

## Patients and methods

Orton Orthopaedic Hospital is a private hospital specializing in joint arthroplasty and spine surgery. In 2012, Orton launched its own treatment registry for degenerative spine disorders. At that time, there was no national spine registry in Finland. The Swedish National Register (Swespine) was chosen as the registry model. The results of the first 5-year follow-up were completed in 2017.

The target group of the study was a series of consecutive patients who underwent elective LSS surgery at Orton Orthopaedic Hospital between 2012 and 2018 and returned follow-up forms within the first or second year. Participation in the registry follow-up was voluntary. In total, 508 LSS decompression surgeries were performed. Based on the aforementioned criteria, a total of 359 (71%) patients were selected, of whom 163 were male (45%) and 196 were female (55%). Mean age was 68.9 years (age range 17–93 years). The baseline characteristics of the population are presented in Table 1.

**Table 1:** Baseline characteristics of the study group

	Normal weight < 25 *N* = 106	Overweight 25.0–29.9 *N* = 154	Obese ≥30 *N* = 99	*P* value
BMI, mean (SD)	22.7 (1.9)	27.5 (1.4)	33.8 (3.2)	..
Female, *n* (%)	63 (59)	78 (51)	55 (56)	0.37
Age, mean (SD)	70 (12)	70 (10)	67 (13)	0.063
Diagnosis, *n* (%)				0.31
Central spinal stenosis without olisthesis (≤ 3 mm)	58 (55)	95 (62)	63 (64)	
Central spinal stenosis with olisthesis (> 3 mm)	36 (34)	38 (25)	21 (21)	
Lateral stenosis	12 (11)	21 (14)	15 (15)	
Duration of back pain, *n* (%)				0.86
No pain	5 (5)	5 (3)	2 (2)	
Less than 3 months	7 (7)	10 (7)	4 (4)	
3–12 months	27 (26)	35 (24)	28 (29)	
1–2 years	17 (16)	22 (15)	17 (17)	
Over 2 years	49 (47)	72 (50)	47 (48)	
Duration of leg pain, *n* (%)				0.71
No pain	3 (3)	4 (3)	3 (3)	
Less than 3 months	9 (8)	10 (7)	7 (7)	
3–12 months	32 (30)	47 (31)	28 (29)	
1–2 years	26 (25)	32 (21)	19 (19)	
Over 2 years	36 (34)	59 (39)	41 (42)	
Smoker, *n* (%)	12 (11)	19 (13)	8 (8)	0.56
VAS, mean (SD)				
Back	52 (28)	55 (26)	57 (28)	0.49
Leg	56 (26)	61 (25)	60 (25)	0.28
Regular pain medication, *n* (%)	56 (53)	80 (52)	55 (56)	0.85
Walking distance < 0.5 km	62 (58)	96 (62)	82 (83)	0.007

Patients were grouped into three based on their body mass index (BMI): normal weight (BMI < 25), overweight (BMI 25–29.9), and obese (BMI ≥ 30). The normal weight group had 106 patients (29%), overweight had 154 patients (43%) and obese had 99 patients (28%).

Prior to surgery, all patients visited an orthopaedic spine surgeon for a preoperative visit. The patient's height and weight, age, gender, smoking status and walking distance were documented. The duration and intensity of back and leg pain were determined using the Visual Analogue Scale (VAS). The intensity of pain experienced was measured on a 0–10 scale with a value of 0 meaning no pain and a value of 10 indicating the worst possible pain. Walking distance was divided into three subgroups: walking distance ranging from 0 to 0.5 km, 0.5 to 1 km and ≥ 1 km. As walking distance generally decreases in LSS, the cut-off value for walking distance was 0.5 km.

Information on QoL and functional disability before and after surgery was collected using the EQ-5D [9] and ODI questionnaires [10]. The EQ-5D is a five-dimensional survey in which each dimension (mobility, self-care, everyday activities, pain/discomfort, and depression/anxiety) is given a value of 1–3. Combining these points gives a five-digit score, where a higher value means a better quality of life.

The ODI questionnaire is used to assess the functional impairment caused by back pain. A score below 20% indicates a mild disability, while a score above 40% indicates a severe functional limitation.

Spine surgeries were performed by microscopic partial laminectomy, removal of the thickened ligamentum flavum, and subarticular decompression. Additional arthrodesis was performed if there were findings suggestive of spinal instability.

Follow-up data for perceived treatment success and QoL at 1 and 2 years were collected using EQ-5D and ODI questionnaires that were sent to the patient.

Data was processed according to the Swespine model (Svensk Ryggkirurgisk Förening). Central spinal stenosis is classified into two groups based on whether it is with or without olisthesis due to the old format of the original Swespine-registry. In this research study, central and lateral stenoses are treated as one diagnostic group. Level of compression, radiographic severity of stenosis and possible arthrodesis are not separately analysed.

### Stat

The descriptive statistics were presented as means with standard deviation (SD or counts with percentages). Group differences in the baseline were investigated through a series of one-way analysis of variances (ANOVA) and chi-square test. Repeated measures of the changes in outcomes were compared between BMI-level groups with mixed-effect models and an unstructured covariance structure (i.e. the Kenward-Roger method for calculating the degrees of freedom). Fixed effects included the group, the time, and group X time interactions. We used age and gender as covariates when appropriate. The repeated measurements were taken at different time points, including baseline, 0, 1 and 2 years. Mixed models allowed the analyses of unbalanced datasets without imputation; therefore, we analyzed all available data with the full analysis set. Normal distributions were evaluated graphically and with the Shapiro–Wilk test. All analyses were performed with Stata 16.1 (StataCorpLP; College Station, TX, USA).

## Results

The mean BMI in the normal weight group was 22.7 (SD 1.9), in the overweight group 27.5 (SD 1.4) and in the obese group 33.8 (SD 3.2). The preoperative mean VAS in the back and leg in normal weight group was 52 (SD 28) and 56 (SD 26), in the overweight group 55 (SD 26) and 61 (SD 25) and in the obese group 57 (SD 28) and 60 (25), respectively.

Preoperative baseline measurements (Table 1) showed no significant differences between the BMI groups, except in walking distance where those patients with a BMI ≥25 were associated with a shorter walking distance (*p* = 0.007).

Preoperatively, patients with a BMI ≥ 30 had an inferior QoL, as measured by the EQ-5D questionnaire, but showed no statistical difference (*p* = 0.41). Generally, all groups had improved QoL following an LSS operation, but changes over 2 years showed no statistical difference in clinical outcomes (*p* = 0.92) (Table 2).

**Table 2:** Changes in EQ-5D and ODI over 2 years following lumbar spinal stenosis (LSS) surgery

	Before surgery			*P* value*	Change over 2 years			*P* value*
	BMI				BMI			
	Normal < 25	Overweight 25.0–29.9	Obese ≥30		Normal < 25	Overweight 25.0–29.9	Obese ≥30	
	Mean (±SD)	Mean (±SD)	Mean (±SD)		Mean (95% CI)	Mean (95% CI)	Mean (95% CI)	
EQ-5D	0.49 (0.32)	0.48 (0.29)	0.43 (0.28)	0.41	0.23 (0.17 to 0.29)	0.22 (0.17 to 0.27)	0.22 (0.16 to 0.29)	0.92
ODI	28 (21)	30 (20)	36 (20)	0.001	− 17 (− 21 to − 14)	− 15 (− 18 to − 10)	− 14 (− 18 to − 10)	0.54

*Adjusted for age and gender. *P* value represents change over 2 years in different BMI groups

*EQ-5D* EuroQol-5D, *ODI* Oswestry Disability Index

Functional disability due to back pain, as measured by the Oswestry Disability Index (ODI), was preoperatively statistically higher in patients with a BMI ≥ 30 (*p* < 0.001). At 2-year follow-up, all groups had improved ODI scores, but there was no statistical difference with the BMI groups (*p* = 0.54).

**Fig. 1:**
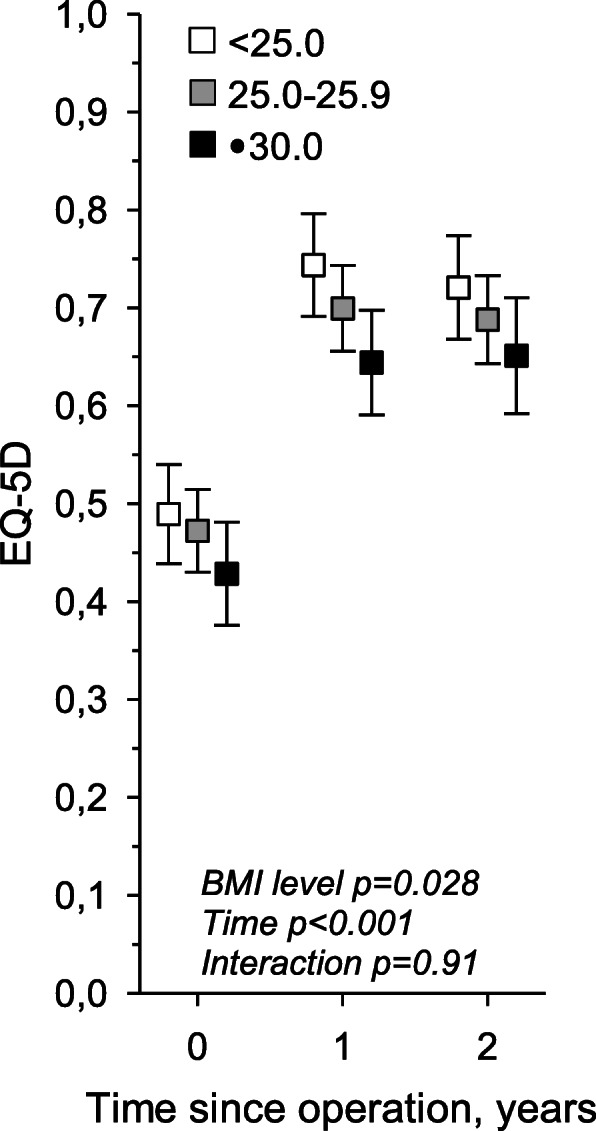
Change in EQ-5D over 2 years following lumbar spinal stenosis (LSS) surgery. Values were adjusted for age and gender

In Fig. 1, both BMI levels and time following operation showed statistical significance (*p* = 0.028 and *p* < 0.001, respectively).

## Discussion

The results of the LSS surgeries suggest that normal weight, overweight, and obese patients with LSS all benefited from operative treatment. Preoperatively, patients with a BMI ≥ 30 had an inferior QoL, as measured by the EQ-5D questionnaires. One year after the operation, all groups had a similar trend of improved QoL. At the second year, the results in all groups levelled off. There was no statistical difference in clinical outcomes.

Functional disability due to back pain, as measured by ODI, was preoperatively statistically higher in those patients with a BMI ≥ 30 than in other BMI groups. Mean ODI for obese patients was 36, while for normal weight patients it was 28. After a 2-year follow-up, all groups had improved ODI scores, meaning they had a benefit from the operation, but the change in ODI was not significant between the groups. The mean change for obese patients was − 14, while for patients with a normal weight it was − 17. In a cohort study of Swedish spine operations, Knutsson et al. [11] found that at a 2-year follow-up following LSS surgery, obese patients had higher ODI scores compared with normal weight patients.

Similar to our results, Giannadakis et al. [6] found that both non-obese and obese patients reported considerable clinical disability improvement 1 year after LSS surgery.

Elsayed et al. [12] suggested that obese patients may need longer recovery times after decompression surgery but tend to reach equivalent results as patients that are overweight or of normal weight in a one-year follow-up. Obese patients had higher leg pain and ODI scores three months after LSS surgery, but the difference in patient-related outcome measures (PROMs) disappeared by 12 months.

A strength of this study was the relatively large sample size and the different baseline characteristics of the study group that were taken into consideration. Preoperative baseline measurements (Table 1) showed no significant differences between the BMI groups, except in walking distance where patients with a BMI ≥ 25 were associated with a shorter walking distance.

Another strength of this series was the retrospective longitudinal evaluation of the change in QoL and functional disability at the surgery time and 2 years after that. However, compared with randomized clinical trials, there is a possibility of selection bias due to the selection of patients from a disease registry and thus lower external validity of results to other populations. This deficiency is present in all retrospective research studies.

One limitation of this study is that postoperative complications were not clearly categorized in the Finspine registry. Obesity has generally been linked to a higher risk of significant postoperative complications in spine surgery [7]. Most literature involving obesity and postoperative complication rates with LSS surgery include the addition of arthrodesis to neural decompression and so are not directly comparable. Rihn et al. [8] found that obesity is not associated with a worse clinical outcome or a higher rate of postoperative complications following LSS surgery.

Prior to the operation, obese patients should be motivated to lose weight and effectively treat other comorbidities to lower the risk of postoperative complications relating to any type of surgical treatment. Currently, research studies suggest that obesity predisposes patients to a higher risk of postoperative complications when arthrodesis is combined with decompression.

Another limitation of this study was that the severity of stenosis, level of spinal stenosis, and possible earlier spine operations were not analysed. Moreover, the addition of arthrodesis to decompression was not separately evaluated. Kuittinen et al. [13] found that severe central stenosis and one-level central stenosis predict a positive outcome of LSS surgery, while multilevel stenosis is associated with a negative outcome. Herno et al. [14] found that patients with previous back surgery had a significant negative impact on the outcome of reoperation for LSS.

Confounding variables such as social situation, together with psychological and somatic comorbidities, were not evaluated. The Finspine registry used for data collection and processing does not include selection criteria for other comorbidities. In the future, the spine registry should be developed to include more precise diagnoses to be better able to evaluate the importance of psychological and somatic comorbidities in surgical outcomes. They can also be used by surgeons preoperatively to optimize individual treatment plans.

The research findings of this study suggest that surgical intervention for debilitating or longstanding symptoms of LSS should be considered as a treatment option for suitable patients in spite of their BMI.

## Data Availability

The datasets generated and/or analysed during the current study are not publicly available due the registry study data but are available from the corresponding author on reasonable request.

## Abbreviations

LSS: Lumbar spinal stenosis
QoL: Quality of life
ODI: Oswestry Disability Index
EQ-5D: EuroQol five-dimension scale
BMI: Body mass index
VAS: Visual analogue scale
ANOVA: Analysis of variance
